# Supplementary material for: Growth Hormone-Releasing Hormone (GHRH) Antagonist Peptides Combined with PI3K Isoform Inhibitors Enhance Cell Death in Prostate Cancer
Source: Cancers (Basel). 2025 May 13;17(10):1643. doi: 10.3390/cancers17101643 (PMC12110010; doi:10.3390/cancers17101643)
Supplement: Supplementary file 1 [file cancers-17-01643-s001.zip › cancers-3558218-supplementary.pdf]

Supplementary Figures 1-9
Supplementary Table 1

Growth Hormone-Releasing Hormone (GHRH) Antagonist Peptides Combined with PI3K Isoform Inhibitors Enhances Cell Death in Prostate Cancer

Carlos Perez-Stable, Alicia de las Pozas, Medhi Wangpaichitr, Wei Sha, Haibo Wang, Renzhi Cai, Andrew V. Schally

Supplementary Figure S1

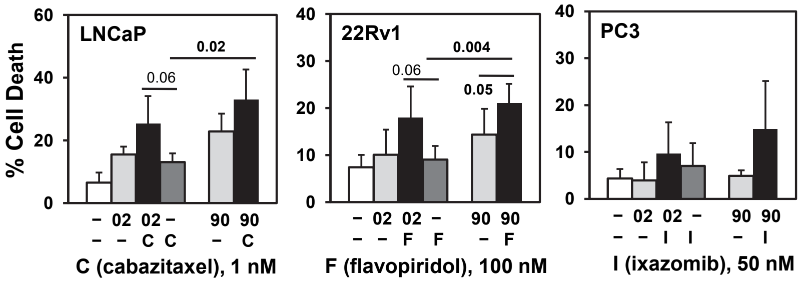

Figure S1. Searching for a drug combination with MIA-602 and -690 that will increase cell death in PCa/CRPC/NEPC. Trypan blue exclusion assay showed the anti-mitotic cabazitaxel (C, 1 nM) + MIA-602 (02) or -690 (90) (5 µM) did not significantly increase cell death in LNCaP compared to C, 02/90, and control cells. The anti-CDK drug flavopiridol (F, 100 nM) + MIA-690 (but not MIA-602) increased cell death in 22Rv1. The proteasome inhibitor ixazomib (I, 50 nM) + MIA-602 or -690 did not increase cell death in PC3. *P* values are shown near the bars.

Supplementary Figure S2

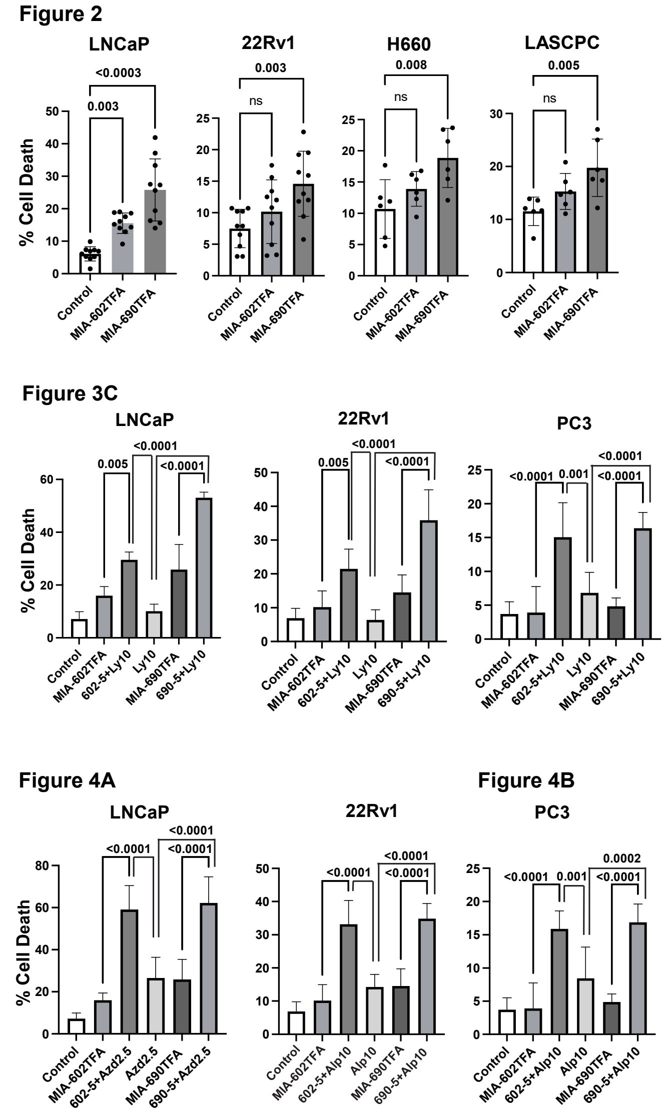

Figure S2. Recalculation of cell death (trypan blue) data using ANOVA. Top panel: data from Fig. 2 recalculated using one-way ANOVA followed by Dunnett's multiple comparisons (GraphPad). Middle panel: data from Fig. 3C recalculated using one-way ANOVA followed by Šidáks multiple comparisons test. Ly10 refers to pan-PI3K inhibitor LY294002 (10 µM). Bottom panel: data from Fig. 4A, B recalculated using one-way ANOVA followed by Šidáks multiple comparisons test. Azd2.5 refers to PI3Kβi (2.5 µM) and Alp10 refers to PI3Kαi (10 µM). *P* values are shown on top of bars. Not significant (ns).

Supplementary Figure S3

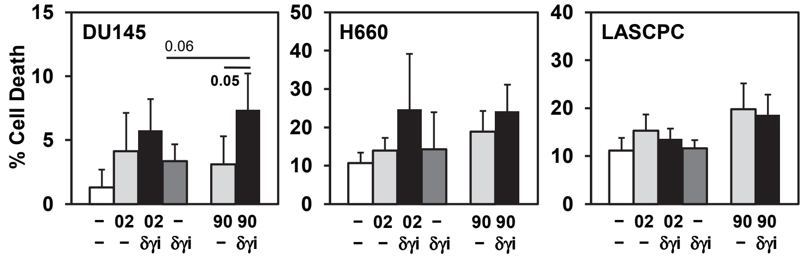

Figure S3. PI3Kδ/γ inhibitor (δγi, duvelisib) does not increase MIA-602 and -690 cell death. Trypan blue exclusion assay showed δγi (10 μM) + MIA-602 (02) or -690 (90) (5 μM) did not increase cell death in DU145, H660, or LASCPC. *P* values are shown near the bars.

Supplementary Figure S4

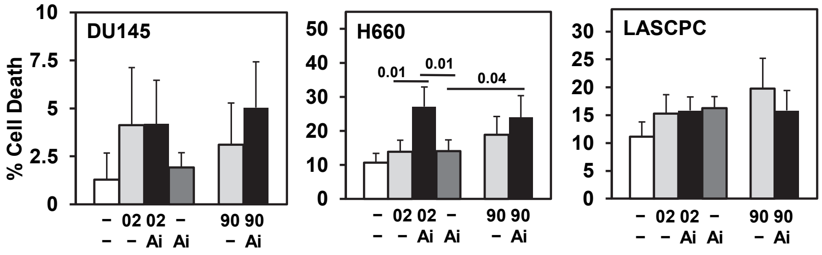

Figure S4. AKT inhibitor (Ai, capivasertib) does not increase MIA-602 and -690 cell death. Trypan blue exclusion assay showed Ai (10 µM DU145; 25 nM H660/LASCPC) + MIA-602 (02) or -690 (90) (5 µM) did not increase cell death in DU145, H660 (except Ai + 02), or LASCPC. *P* values are shown near the bars.

Supplementary Figure S5

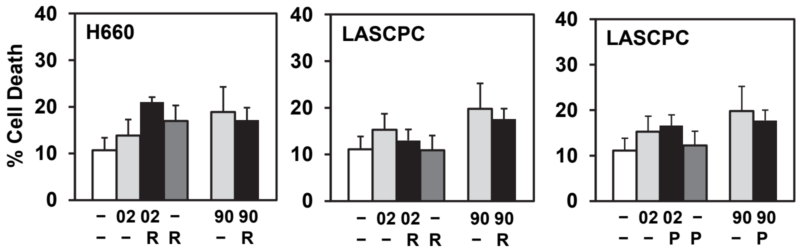

Figure S5. mTOR and NFκB inhibitors do not increase MIA-602 and -690 cell death in NEPC cells. Trypan blue exclusion assay showed mTOR inhibitor rapamycin (R, 0.05 nM) or NFκB inhibitor parthenolide (P, 0.5 μM) + MIA-602 (02) or -690 (90) (5 μM) did not increase cell death in NEPC cells H660 and LASCPC. mTOR is highly activated in NEPC cells.

Supplementary Figure S6

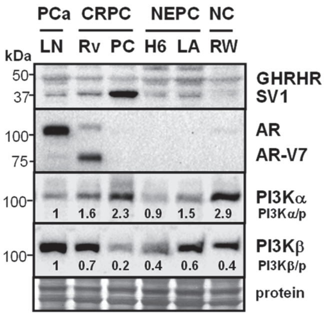

Figure S6. Comparison of PI3Kα and PI3Kβ protein levels in PCa/CRPC/NEPC cells. Western blot analysis showed PI3Kα protein was highest in PC3 (PC) compared to LNCaP (LN) whereas PI3Kβ protein was highest in LNCaP compared to PC3, H660 (H6), and LASCPC (LA). 22Rv1 (Rv) had intermediate levels of PI3Kα and PI3Kβ protein. For comparison, RWPE-1 (RW), a non-cancer (NC) prostate epithelial cell line, showed high PI3Kα and lower PI3Kβ. Also shown were protein levels of GHRHR (high splice variant 1 [SV1] in PC3) and AR (highest in LN, variant 7 [AR-V7] in Rv, weak in RW, and negative in PC, H6, LA). Protein refers to Coomassie blue stain of blots after all immunological analysis was completed. Quantification values (divided by protein [p]) for PI3Kα and PI3Kβ were shown below specific bands with LN value =1.

Supplementary Figure S7

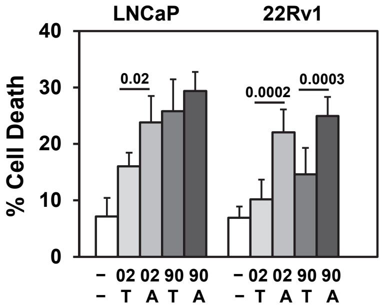

Figure S7. MIA-602Ac and -690Ac are better at increasing cell death in 22Rv1 compared to MIA-602/690TFA. Trypan blue exclusion assay showed MIA-602/690Ac (A, 5 μM) significantly increased cell death in 22Rv1 better than MIA-602/690TFA (T, 5 μM). In LNCaP, MIA-602Ac significantly increased cell death compared to MIA-602TFA; no difference was noted between MIA-690Ac and MIA-690TFA. *P* values are shown above the bars.

Supplementary Figure S8

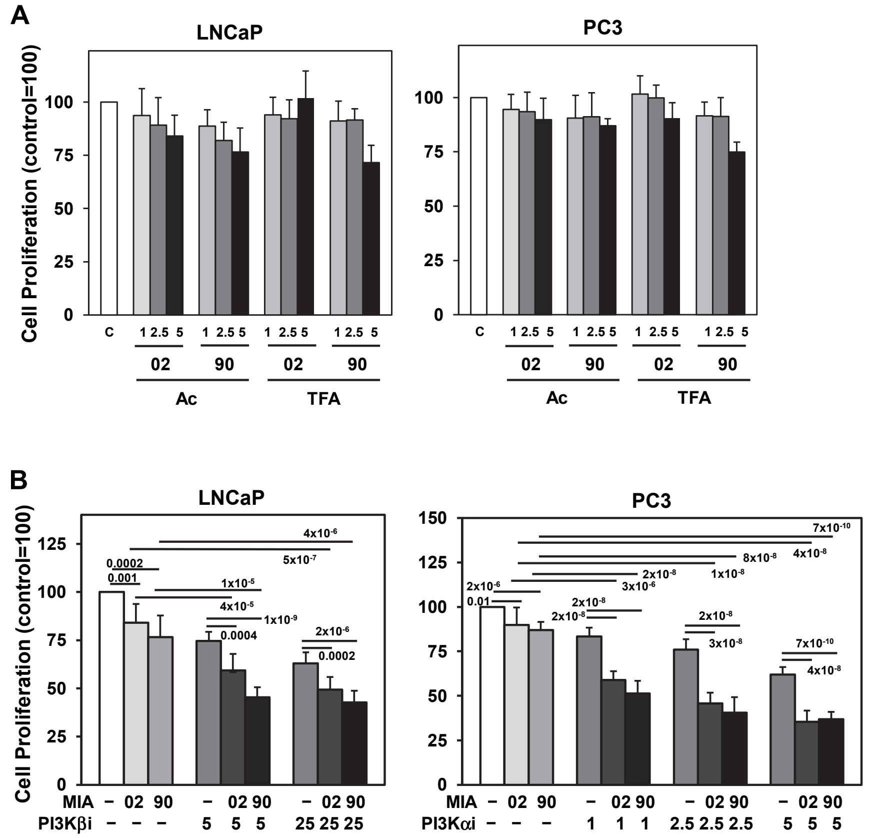

Figure S8. MIA-602/690Ac + PI3K inhibitors significantly decreases cell proliferation in LNCaP and PC3. (**A**) Cell proliferation assay (72h) showed 602/690 (02, 90) in Ac or TFA forms have little or modest effects on LNCaP and PC3. Concentrations used were 1, 2.5, and 5 µM. (**B**) Cell proliferation assay (72h) showed the combination of MIA-602/690Ac (5 µM) + PI3Kβi (5, 25 nM) significantly decreased LNCaP proliferation compared to each alone and control (=100). In PC3, MIA-602/690Ac + PI3Kαi (1, 2.5, 5 µM) also significantly decreased proliferation compared to each alone. *P* values are shown near the bars.

Supplementary Table S1

MIA602/690Ac + PI3K isoform inhibitors synergistically inhibits LNCaP and PC3

LNCaP

602 (μM)	FA	PI3Kβi (nM)	FA	FA (602+βi)	CI
5	0.16	5	0.25	0.41	0.26
5	0.16	25	0.37	0.51	0.27

690 (μM)	FA	PI3Kβi (nM)	FA	FA (690+βi)	CI
5	0.24	5	0.25	0.55	0.11
5	0.24	25	0.37	0.57	0.16

PC3

602 (μM)	FA	PI3Kαi (μM)	FA	FA (602+αi)	CI
5	0.10	1	0.17	0.41	0.25
5	0.10	2.5	0.24	0.54	0.31
5	0.10	5	0.38	0.65	0.37

690 (μM)	FA	PI3Kαi (μM)	FA	FA (690+αi)	CI
5	0.13	1	0.17	0.49	0.15
5	0.13	2.5	0.24	0.60	0.23
5	0.13	5	0.38	0.63	0.39

Cell proliferation assay (72h) showed MIA-602Ac or -690Ac (5 μM) + various combinations PI3Kβi (5, 25 nM; LNCaP) or PI3Kαi (1, 2.5, 5 μM; PC3) inhibited LNCaP and PC3 (strong synergy), as determined by combination index (CI). FA, fraction affected refers to inhibition (no inhibition control=0; 100% inhibition= 1.0).

Supplementary Figure S9

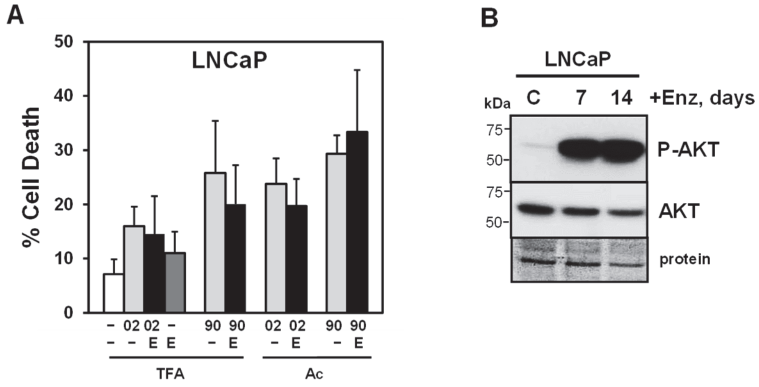

Figure S9. AR antagonist enzalutamide does not increase MIA-602 and -690 cell death in LNCaP cells. (**A**) Trypan blue exclusion assay showed enzalutamide (E, 10 μM) + MIA-602 or -690 (TFA or Ac; 5 μM) did not increase cell death compared to 602/690 alone in LNCaP. (**B**) Western blot analysis showed treatment of LNCaP with enzalutamide (Enz) for 7 or 14d highly increased P-AKT in LNCaP whereas total AKT remained the same. Protein refers to Coomassie blue stain of blots after all immunological analysis was completed.
